# Genome wide association and linkage analyses identified three loci—4q25, 17q23.2, and 10q11.21—associated with variation in leukocyte telomere length: the Long Life Family Study

**DOI:** 10.3389/fgene.2013.00310

**Published:** 2014-01-17

**Authors:** Joseph H. Lee, Rong Cheng, Lawrence S. Honig, Mary Feitosa, Candace M. Kammerer, Min S. Kang, Nicole Schupf, Shiow J. Lin, Jason L. Sanders, Harold Bae, Todd Druley, Thomas Perls, Kaare Christensen, Michael Province, Richard Mayeux

**Affiliations:** ^1^Sergievsky Center, College of Physicians and Surgeons, Columbia UniversityNew York, NY, USA; ^2^Taub Institute, College of Physicians and Surgeons, Columbia UniversityNew York, NY, USA; ^3^Department of Epidemiology, School of Public Health, Columbia UniversityNew York, NY, USA; ^4^Department of Neurology, College of Physicians and Surgeons, Columbia UniversityNew York, NY, USA; ^5^Division of Statistical Genomics, Department of Genetics, Washington University School of MedicineSt. Louis, MO, USA; ^6^Department of Epidemiology, University of PittsburghPittsburgh, PA, USA; ^7^Department of Human Genetics, University of PittsburghPittsburgh, PA, USA; ^8^Center for Aging and Population Health, University of PittsburghPittsburgh, PA, USA; ^9^Department of Psychiatry, College of Physicians and Surgeons, Columbia UniversityNew York, NY, USA; ^10^Department of Biostatistics, Boston University Medical CenterBoston, MA, USA; ^11^Department of Pediatrics and Genetics, Washington University School of MedicineSt. Louis, MO, USA; ^12^Department of Medicine, Boston University Medical CenterBoston, MA, USA; ^13^The Danish Aging Research Center, Epidemiology, University of Southern DenmarkOdense, Denmark

**Keywords:** telomere length, aging, familial longevity, genome wide association and linkage, family-based study, novel genes

## Abstract

Leukocyte telomere length is believed to measure cellular aging in humans, and short leukocyte telomere length is associated with increased risks of late onset diseases, including cardiovascular disease, dementia, etc. Many studies have shown that leukocyte telomere length is a heritable trait, and several candidate genes have been identified, including *TERT, TERC, OBFC1, and CTC1*. Unlike most studies that have focused on genetic causes of chronic diseases such as heart disease and diabetes in relation to leukocyte telomere length, the present study examined the genome to identify variants that may contribute to variation in leukocyte telomere length among families with exceptional longevity. From the genome wide association analysis in 4,289 LLFS participants, we identified a novel intergenic SNP rs7680468 located near *PAPSS1* and *DKK2* on 4q25 (*p* = 4.7E-8). From our linkage analysis, we identified two additional novel loci with HLOD scores exceeding three, including 4.77 for 17q23.2, and 4.36 for 10q11.21. These two loci harbor a number of novel candidate genes with SNPs, and our gene-wise association analysis identified multiple genes, including *DCAF7, POLG2, CEP95, and SMURF2* at 17q23.2; and *RASGEF1A, HNRNPF, ANF487, CSTF2T, and PRKG1* at 10q11.21. Among these genes, multiple SNPs were associated with leukocyte telomere length, but the strongest association was observed with one contiguous haplotype in *CEP95* and *SMURF2*. We also show that three previously reported genes—*TERC, MYNN, and OBFC1*—were significantly associated with leukocyte telomere length at *p*_empirical_ < 0.05.

## Introduction

Telomere shortening is a marker of *in vivo* cellular aging, and leukocyte telomere length is related to life span (Holt et al., 1996; Chadwick and Cardew, 1997; Shay and Wright, 2001; Aviv et al., 2006; Christensen et al., 2006; Armanios and Blackburn, 2012). Individuals with short leukocyte telomere length are at an increased risk of age-related diseases (e.g., cardiovascular diseases, diabetes, dementia, cancer) and premature subsequent death compared with similarly aged individuals with longer telomeres (Jeanclos et al., 1998; Epel et al., 2004; Aviv, 2009, 2012; Kaplan et al., 2009; Honig et al., 2012; Shaffer et al., 2012; Ye et al., 2013). However, the direction and strength of association between leukocyte telomere length and the risk of these diseases vary across studies (Aviv et al., 2006; Sanders et al., 2012).

Both longevity and leukocyte telomere length are heritable traits with estimates ranging from 20 to 40% (Perls et al., 1998, 2000, 2007; Cournil and Kirkwood, 2001; Perls and Terry, 2003; Lee et al., 2004; Beekman et al., 2006; Christensen et al., 2006; Hjelmborg et al., 2006; Deelen et al., 2011; Newman et al., 2011; Murabito et al., 2012) and 34–82% (Aulchenko et al., 2004; Vasa-Nicotera et al., 2005; Broer et al., 2013), respectively. As with most complex traits, multiple genetic variants along with environmental and lifestyle factors are likely to contribute to familial longevity. Genome wide linkage studies have identified four chromosomal regions, 14q23.2, 10q26.13, and 3p26.1 (Andrew et al., 2006), and 12p11 (Vasa-Nicotera et al., 2005) that may harbor loci that influence leukocyte telomere length. Genome wide association studies (GWAS), and meta-analysis across several studies in multiple populations have revealed several candidate genes: *TERC*, telomerase RNA component (Codd et al., 2010), located on 3q26 (Soerensen et al., 2012), and *TERT*, telomerase reverse transcriptase (5p15.33) (Hartmann et al., 2009; Soerensen et al., 2012). A meta-analysis (Mangino et al., 2012) identified *CTC1* (conserved telomere maintenance component 1, 17p13.1) and *ZNF676* (zinc finger protein 676, 19p12) as candidate genes for telomere homeostasis in humans, and confirmed that minor variants of *OBFC1* on 10q24.33 was associated with shorter leukocyte telomere length. Although their function is not certain, these genes appear to be involved in maintenance of chromosome structures.

To identify genetic variants contributing to variation in leukocyte telomere length, we analyzed data from a large cohort of families that had multiple family members who survived to exceptionally old age (the Long Life Family Study). To detect both common and rare variants that contribute to leukocyte telomere length, this study applied two different approaches: family based association analysis and joint linkage and association analysis. In addition, a heterogeneity model for linkage analysis was applied to account for possible genetic heterogeneity across families since different families may achieve longevity through different means.

## Materials and methods

### Study design and subjects for the primary study

We employed a 2-stage genome wide study in genotyping participants from of the Long Life Family Study (LLFS; http://www.longlifefamilystudy.org). The details of study design and protocols are described by Newman et al. (2011). Briefly, the LLFS is a multicenter study with recruitment from four centers, Boston University Medical Center, Boston, MA, Columbia University Medical Center, New York, NY; University of Pittsburgh, Pittsburgh, PA; and University of Southern Denmark, Odense, Denmark. Nearly 5,000 Caucasian subjects from families with strong evidence for familial longevity had been recruited and examined (Sebastiani et al., 2009). The ascertainment strategy for the Denmark cohort differed slightly from US sites. Individuals who were at least 90 years of age during the study recruitment period were identified in the Danish National Register of Persons (Pedersen et al., 2006). Using the parental information on birth place, names, and parish registers available in regional archives, sibships were identified and, potentially eligible families were identified and contacted to assess the family's eligibility and willingness to participate in the LLFS using the criteria parallel to those used in the US. Of those, 4289 individuals from 586 families had measures of leukocyte telomere length and were included in the genome wide association and linkage analyses.

### Covariates

Information on demographic and medical history information was obtained from participants by self-report (Newman et al., 2011). To assess potential confounding effects, multiple covariates were included in a polygenic model of heritability analysis in SOLAR (Almasy and Blangero, 1998, 2010): age, sex, education, site, generation, smoking (ever vs. never), alcohol consumption (yes vs. no), marital status (widowed/divorced vs. never married vs. married), a history of heart disease (yes/no) and diabetes (yes/no), and 20 principle components. In all subsequent analyses, we included covariates that were significant at *p* < 0.05: age, sex, education, site, smoking, alcohol consumption, marital status, a history of heart disease, and principle component 8 (PC8). Even though not all three indicator variables for site were significant, we forced site variables into the model.

### Measurements of leukocyte telomere length

Assays of average leukocyte leukocyte telomere length were performed using our modification of a method developed by Cawthon et al. (Cawthon, 2002; Cawthon et al., 2003). Briefly, the coded DNA samples were processed by laboratory personnel, blinded to participant characteristics. Real-time PCR was performed using a CFX384 thermocycler (Biorad, Richmond, CA). Assay method was optimized for use of both telomere (T) and single copy gene (S) amplifications on the same 384-well plate, with reference standard DNA samples on each plate. Test DNA samples each underwent two triplicate PCR reactions, with use of “calibrator samples” for correction for inter-plate variability. Amplification primers for telomeres included T_for_: 5′- CGGTTTGTTTGGGTTTGGGTTTGGGTTTGGGTTTGGGTT-3′ and T_rev_: 5′- GGCTTGCCTTACCCTTACCCTTACCCTTACCCTTACCCT-3′, and for single copy gene (beta-globin) S_for_ 5′- GCTTCTGACACAACTGTGTTCACTAGC-3′ and S_rev_ 5′- CACCAACTTCATCCACGTTCACC-3′. Thermocycling parameters were 95°C × 10 min activation, followed by 34 cycles of 95°C × 15 s, and 55°C × 120 s. Our assay coefficient of variance was 5–8%. T/S ratio was converted to basepairs (bp) leukocyte telomere length by use of the linear regression formula: *bp* = (1,585 * T/S ratio) + 3582, obtained by co-analysis of selected DNA samples using both PCR and terminal restriction fragment (non-radioactive *T*elo*TAGGG* leukocyte telomere length, Roche Diagnostics, Mannheim, Germany) methods (correlation coefficient *r* = 0.90).

**Figure A1:**
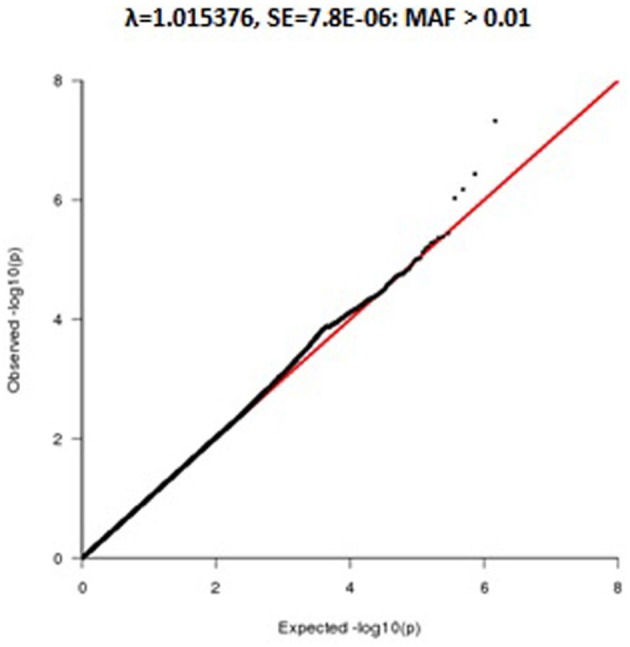
**Q-Q Plot of telomere association using Inverse—normal transformation**.

Because of non-normality of the leukocyte telomere length distribution (skewness = 2.22 and kurtosis = 12.19), we transformed leukocyte telomere length using an inverse normal function to minimize potential inflation of type 1 error rates (Allison et al., 1999; Etzel et al., 2003). Following the transformation, the distribution of leukocyte telomere length conformed to a normal distribution (skewness = −0.0078 and kurtosis = −0.0489) in all family members (See Figure A1).

### Genotyping

SNP Chips manufactured by Illumina (Human Omni 2.5 v1) were used by the Center for Inherited Disease Research (CIDR) for genotyping. In depth Quality control (QC)-process was carried out in the Division of Statistical Genomics, Washington University in Saint Louis. QC was performed before imputation and included assessment of Mendelian errors as implemented in LOKI (Heath, 1997) and verification of reported pedigree relationships using GRR (Abecasis et al., 2001). 83,774 SNPs with a lower call rate <98% per marker were dropped; in addition, a total of 3,647 SNPs with high Mendelian error rate were dropped. Eighteen subjects who did not reach a 97.5% call rate were dropped. In addition, 153,363 Mendelian errors were set to missing. Of approximately 2.23 M autosomal SNPs, approximately 1.47 M with a minor allele frequency (MAF) >1% were used in the analysis.

### Population structure

To examine underlying population structure, principal components (PCs) analysis was performed as implemented in Eigenstrat (Patterson et al., 2006; Price et al., 2006). SNPs with MAF <5%, Hardy-Weinberg equilibrium (HWE) *p*-value <10^−6^, and with missing genotypes were excluded. In addition, 1613 SNPs from some special regions (2q21, 2q21.1, HLA1, and HLA (chromosome 6), 8p23.1, 8p23, 17q21.31, and 17q21.311) were excluded because of known inversions, HLA and other special regions that may drive the principal component (PC) analysis. After QC procedure, a total of 116,867 tag SNPs were used to create PCs model using 1522 unrelated subjects from LLFS and 361 founders from HapMap data (CEPH: Caucasians, Yoruban: YRI- Africans, Asians: Chinese and Japanese, and Tuscans: TSI- Caucasians) for the same tag SNPs as the ones used for LLFS. The PC model generated 20 PCs, and PC estimators then were expanded, within Eigenstrat framework, to all members of LLFS. PCs were subsequently used as covariates to control for population substructure/admixture.

### Imputation

Imputations were performed based on cosmopolitan phased haplotypes of 1000 Human Genome (1000HG, version 2010–11 data freeze, 2012-03-04 haplotypes; http://www.sph.umich.edu/csg/abecasis/MaCH/download/1000G.2012-03-14.html). Three programs were used: MACH for pre-phasing LLFS data (version 1.0.16); MINIMACH for performing imputations (version of May 2012); and ChunkChromosome script for splitting the LLFS data into smaller blocks to speed the process of imputation. In addition a number of SAS programs were implemented to streamline this process as well as transforming the final data into SAS datasets. Imputations were performed in chunks with 5000 SNPs blocks and 1000 SNPs overlap from our data. A number of filters before imputing were implemented in the LLFS genotypic data by removing markers that had MAF <1%, HWE *p*-value <10^−6^, if LLFS SNPs alleles mismatched with those of 1000HG, and not present in the 1000HG panel, as well as flipping any SNP when appropriate to the forward strand. A total of 38.05 M SNPs were imputed. For single variant-single trait association with imputed dosage two additional filters were implemented, the MAF >1% and the *r*^2^ > 0.3 (a quality score from the imputation), which reduced the analysis to 9.25 M variants.

### Heritability analysis

Heritability was estimated to assess how much phenotypic variance was explained by additive genetic variance using maximum likelihood methods as implemented in SOLAR (Blangero and Almasy, 1997; Almasy and Blangero, 1998). Heritability estimates were computed over all family members within the proband and offspring generations.

### Family-based association analysis

To determine whether *common* variants (MAF >1%) contribute to variation in leukocyte telomere length, a family based genome wide association study (GWAS) was performed. For the GWAS, the most parsimonious linear mixed model was used that comprised several covariates, including age, sex, education, site, smoking, alcohol consumption, marital status, a history of heart disease, and PC8. Of 20 PCs, only PC8 was included in the linear mixed model because it was found to be significant at *p* < 0.05 in the multivariate polygenic model described above. This mixed linear model adjusted for relatedness among family members by incorporating the kinship coefficient matrix using the R functions written by Therneau (Therneau et al., 2012). In addition, to confirm previously reported genes, we examined the regions containing the previously reported genes and applied the linear mixed effects model as above, but we ‘shuffled’ the phenotype 500 times to obtain empirical p-values at three different levels to correct for multiple testing: SNP-wise, gene-wise, and then experiment wise *p*-values. To obtain gene wise p-values, we shuffled the phenotypes and computed p-values to establish the distribution of smallest *p*-values for 500 replicates, and then counted the number of replicates that exceeded the nominal *p*-values for the SNPs within a gene. To obtain the experiment wise *p*-value, we then extended the approach we used for gene-wise estimation to include all genes.

### Linkage study

To determine whether *rare* variants that contribute to leukocyte telomere length segregate in families, linkage analysis using haplotype based identity-by-descent (IBD: ZAPLO O'Connell, 2000) was performed. Specifically, sets of up to five tightly linked SNPs within 0.5 cM intervals were haplotyped with ZAPLO, generating a set of SNP “super-loci” spaced ~0.5 cM apart and having greater information content than individual SNPs. From these haplotypes, Loki (Heath, 1997) was used to estimate multipoint IBD in intact pedigrees, which was then imported into SOLAR for variance component linkage analysis. Because different quantitative trait loci (QTLs) influencing leukocyte telomere length are likely to segregate in different families (i.e., genetic heterogeneity), admixture (heterogeneity) LOD scores (HLOD) were completed using SOLAR (Blangero et al., 2001, 2013; Almasy and Blangero, 2010). This algorithm computes HLOD using the algorithm by C. A. B Smith in which the null hypothesis of homogeneity is compared to the hypothesis of heterogeneity (Smith, 1961; Ott, 1983). The admixture linkage analysis identified two linkage peaks—17q23.2 and 10q11.21—that had HLOD exceeding 3 when all families were included in the analysis. When restricted to linked families, HLODs increased to 5.86 for 17q23.2 and 9.69 for 10q11.21 as expected. This finding suggests that subsequent sequencing experiments can be prioritized to include these families.

To identify the most likely candidate genes from a large set of genes under each linkage peak, family-based Sequence Kernel Association Test (SKAT) was performed to identify genes that are associated with leukocyte telomere length (Wu et al., 2011; Chen et al., 2013). This model adjusts for the same set of covariates as in the linear mixed model and also controls for familial correlation by including kinship coefficient in the model. famSKAT tests whether multiple rare variants contribute to phenotypic variation, and does not assume that all rare variants influence phenotypes in the same direction with the same effect size. Taking one step further, we then applied measured genotype analysis to identify genetic variants that were significantly associated with variation in leukocyte telomere length. In addition, haplotype analysis was conducted when contiguous multiple variants were associated with leukocyte telomere length. Haplotype analysis was performed using PLINK (Purcell et al., 2007) and MERLIN (Abecasis et al., 2002), and the resulting haplotypes from two analyses were identical.

## Results

### Descriptive statistics

Of 4289 subjects with telomere assay, 1418 were from the proband generation and the remaining 2871 were from the offspring generation (Table 1). The overall mean age was 70.1 and ranged from 24 to 110. There was a slightly higher proportion of women compared with men (55.1 vs. 44.9%), and the cohort comprised whites primarily. The mean age at blood draw was the youngest for the Danish site and the oldest for the NY sites (67.2 vs. 74.1). When stratified by generation, however, the maximum mean age difference across sites was 1.8 years (range for the proband generation: 89.0–90.8 years; range for the offspring generation: 59.9–61.4 years), pointing to the fact that Danish families have a larger number of offspring. The overall mean years of education was 11.6 years, and differed between cohorts (proband generation = 9.8 vs. offspring generation = 12.5).

**Table 1:** **Demographic and clinical characteristics: restricted to whites**.

	**Overall**	**Boston**	**Denmark**	**New york**	**Pittsburgh**
Subjects	4289	1145	1178	825	1141
Age at blood draw (Range)	70.1 ± 15.7 (24–110)	69.4 ± 15.9 (32–110)	67.2 ± 14.2 (36–104)	74.1 ± 16.3 (24–108)	71.1 ± 15.9 (36–104)
Proband generation (Range)	89.5 ± 6.7 (55–110)	89.5 ± 7.2 (55–110)	90.8 ± 6.3 (64–104)	89.2 ± 6.8 (58–108)	89.0 ± 6.3 (71–104)
Offspring generation (Range)	60.6 ± 8.4 (24–88)	59.9 ± 8.2 (32–88)	61.4 ± 8.4 (36–87)	60.4 ± 8.3 (24–83)	60.3 ± 8.5 (36–87)
Age of married–in's (Range)	64.9 ± 11.9 (24–98)	65.1 ± 12.6 (37–98)	62.6 ± 10.1 (36–94)	68.4 ± 13.5 (24–94)	68.0 ± 12.8 (36–91)
Proband generation (Range)	83.0 ± 7.0 (55–98)	83.2 ± 8.6 (55–98)	83.6 ± 6.6 (64–94)	82.2 ± 6.8 (67–94)	83.1 ± 5.7 (71–91)
Offspring generation (Range)	61.0 ± 8.7 (24–88)	60.8 ± 9.1 (37–88)	61.0 ± 8.4 (36–87)	60.4 ± 9.3 (24–79)	61.5 ± 8.8 (36–80)
Men (%)	1927 (44.9)	509 (44.5)	539 (45.8)	379 (45.9)	500 (43.8)
Education (years)	11.60 ± 3.62	12.55 ± 2.94	9.73 ± 4.32	12.25 ± 3.25	12.11 ± 2.95
% Smoking (0 vs. 1 vs. 2)^*^	57.1/33.4/6.9%	58.8/37.1/3.2%	50.9/31.2/14.0%	53.8/36.0/3.9%	64.2/30.0/5.3%
% Alcohol consumption (0 vs. 1 vs. 2)^**^	47.4/33.2/19.2%	47.2/36.0/16.8%	26.3/36.0/37.6%	54.9/33.9/9.8%	63.8/26.8/9.3%
% Marital status (0 vs. 1 vs. 2)^***^	21.7/12.4/65.9%	20.2/14.4/65.4%	16.4/8.9/74.7%	27.5/13.8/58.1%	24.2/12.8/62.8%
A history of heart disease	8.60%	8.60%	5.70%	11.30%	9.60%

* *0 = never, 1 = past, 2 = current*.

** *0 = never/occasional drinking, 1 = 1–7 per week, 2 = 7 per week*.

*** *0 = widowed, 1 = some form of marriage, 2 = currently married*.

The mean leukocyte telomere length was 5325.3 bp (*SD* = 485.5), and the mean leukocyte telomere length was slightly longer for women compared with men (5356.7 bp vs. 5287.6 bp) (Table 2). The mean leukocyte telomere length was shorter in the proband generation compared with the offspring generation (5170.5 bp vs. 5401.8 bp, respectively; *p* = 5.9E-50). Leukocyte telomere length was slightly shorter for the Danish cohort compared with the US cohort (5216.2 bp vs. 5366.6 bp). When analyzed separately, factors that were significantly associated with leukocyte telomere length at *p* < 0.05 were heart disease, smoking, drinking and marital status. When these factors were included in the multivariable model in SOLAR as well as SPSS (SPSS, 2013), the following variables remained significant at *p* < 0.05: site, sex, marital status, education, drinking, smoking, heart disease, and PC8.

**Table 2:** **Mean leukocyte telomere length by site and by sex and generation**.

	**Over all**		**Boston**		Denmark^*^		**New york**		**Pittsburgh**	
	**Mean BP**	***SD***	**Mean BP**	***SD***	**Mean BP**	***SD***	**Mean BP**	***SD***	**Mean BP**	***SD***
All subjects	5325.3	485.5	5365.3	502.9	5216.2	363.7	5362.7	552.3	5370.8	508.6
Sex										
Men^**^	5286.8	466.4	5359.2	518.5	5167.8	292	5294.9	504	5335.4	508.4
Women	5356.7	498.4	5370.3	490.5	5257	410.5	5420.3	584.6	5398.5	507.4
Generation										
Proband	5170.5	398.9	5161.8	421.9	5157	367.3	5209.6	411.5	5149.7	382
Offspring	5401.8	505.8	5461.4	509.7	5230.6	361.6	5500.6	622.6	5504.1	528.8
Married-in's										
Proband	5165.2	370	5225.6	457.3	5023.5	221.6	5193.4	395.6	5163.5	296.5
Offspring	5336.6	498.5	5466.8	532.6	5217.6	432.1	5431	521.4	5440.3	539.5
Total	5306.1	482.4	5420.8	526.8	5203.8	423.4	5344.6	491.2	5356.4	494.8

* *Mean leukocyte telomere length comparisons differed significantly between Denmark and Boston as well as Denmark and Pittsburgh*.

** *Mean leukocyte telomere length differed between males and females at p < 0.05 for all sites except for Boston*.

### Heritability

The overall heritability of leukocyte telomere length was estimated to be 0.54 (*SE* = 0.034) in this cohort. Sex specific heritability estimates for men and women were highly significant and similar to each other (*h*^2^_men_ = 0.597 ± 0.064, *p* = 1.80E-23 vs. *h*^2^_women_ = 0.521 ± 0.053, *p* = 1.17E-26). When restricted to one generation, the heritability estimate for the proband generation was lower than that for offspring generation (*h*^2^_proband_ = 0.47, *SE* = 0.068 vs. *h*^2^_offspring_ = 0.848, *SE* = 0.54).

### Allelic association

**Figure 1:**
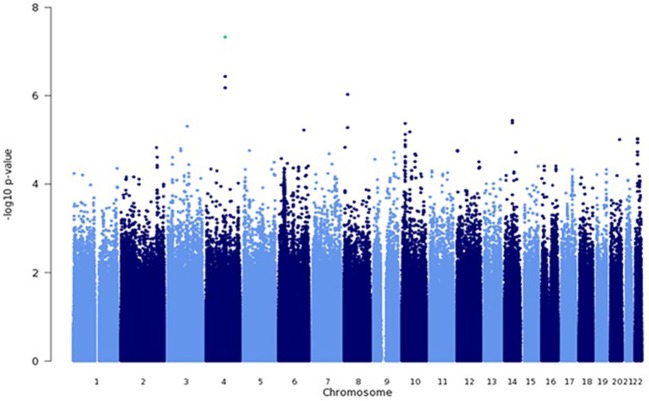
**Genome wide association analysis using a mixed linear model**. GWAS was performed using a mixed linear model that adjusted for age, sex, education, site, smoking, alcohol consumption, marital status, a history of heart disease, and 1 principle component.

Among genotyped SNPs, rs7680468 on chromosome 4q25 reached genome-wide significance (*p* = 4.7E-8) in the LLFS dataset (Figure 1). When imputed SNPs were included, two additional SNPs on 4q25—specifically deletion at c4:108229919 and c4:108229924 — were significantly associated with leukocyte telomere length at *p* < E-8 (Table 3). Altogether five variants, located within or between the *DKK2 and PAPSS1* genes, were associated with telomere length at *p* < 6.6E-7 (Table 3). As these variants span 200 kb, pairwise linkage disequilibrium (D′) for the first three variants ranged from D′ of 0.799 to 0.90, but D′ between SNPs 3 and 4 was 0.25. In addition, several other SNPs were associated with leukocyte telomere length at suggestive levels of significance (*p* <E-6), including SNPs located near genes candidate genes *TMPRSS7* on 3q13.2, *TRDMT1* on 10p13, *SYT16* on 14q23, *TSHZ2* on 20q13.2, and *ASCC2* on 22q12.2 (Table 3).

**Table 3:**
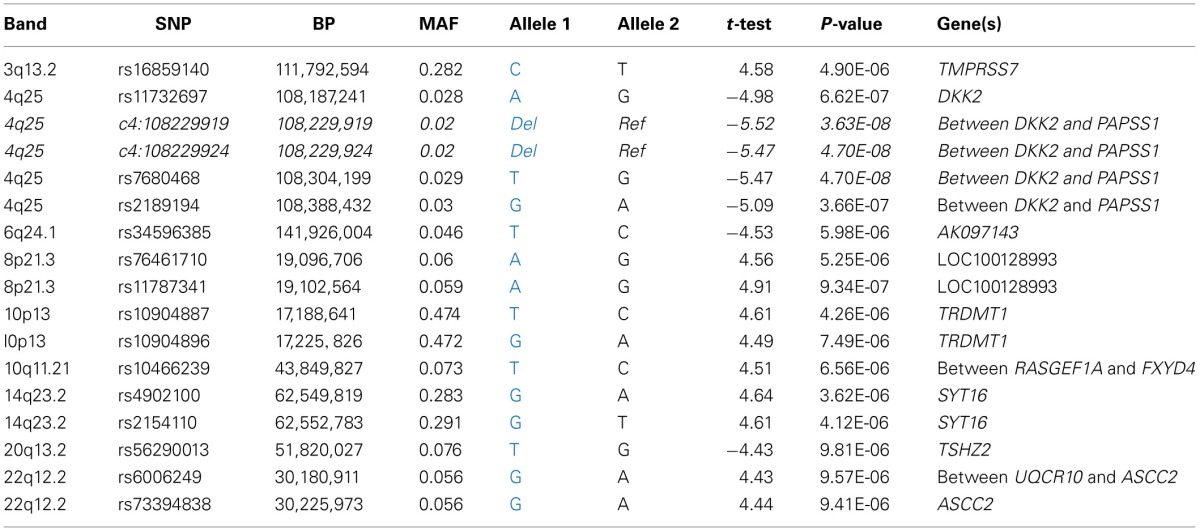
**Candidate SNPs from the genome wide association analysis**.

*BP based on HG19; imputed SNPs are in italic*.

*Minor alleles in the LLFS dataset are in blue*.

### Linkage analysis and measured genotype analysis

**Figure 2:**
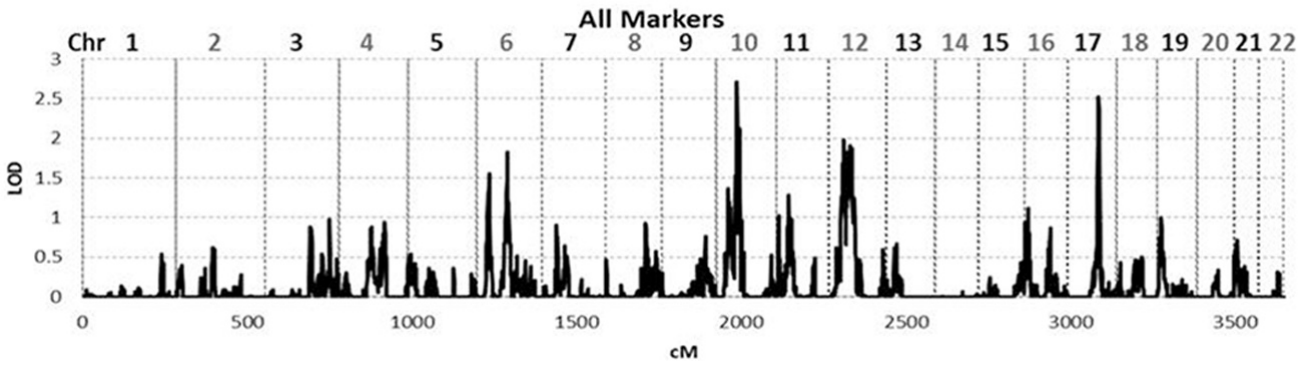
**Genome wide linkage analysis using SOLAR**. Genome wide linkage analysis based on haplotype IBD, adjusting for age, sex, education, site, smoking, alcohol consumption, marital status, a history of heart disease, and 1 principle component.

**Figure 3:**
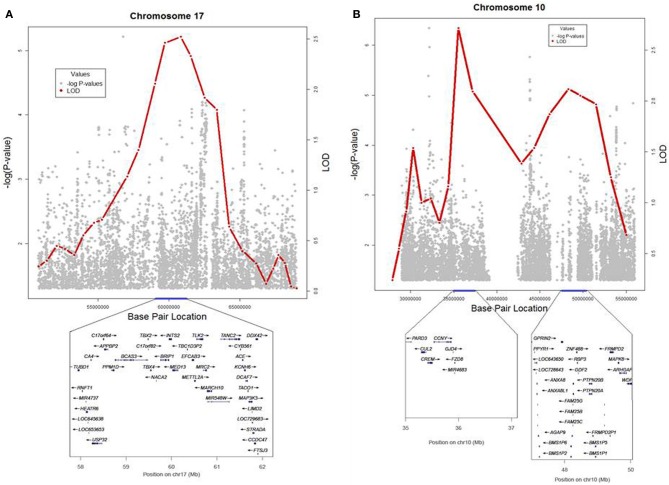
**(A,B)** Locus plots for the results from linkage and association analyses in 17q23.2 and 10q11.21. LOD scores and − log_10_(*p*-value) are presented for the 2 loci.

To identify *rare* variants that segregate in a subset of families that may have been undetected by the linear mixed model, sets of SNP “super-loci” spaced ~0.5 cM apart were used to obtain greater information content than individual SNPs. This linkage analysis based on SOLAR identified four suggestive linkage peaks, including 17q23.2 (LOD = 2.52), 10q11.21 (LOD = 2.72), 12p12.1 (LOD = 1.98), and 6q14.1 (LOD = 1.83) (Figure 2). To assess heterogeneity across families, we computed HLODs for these four loci using all families. HLODs were increased to 4.77 for 17q23.2, 4.36 for 10q11.21, 2.05 for 6q14.1, and 1.99 for 12p12.1 (LOD = 1.98; Table 4). Thus, we subsequently focused on the two loci with HLOD >3 (17q23.2 and 10q11.21), which included multiple candidate genes as shown in Figures 3A,B. To narrow down the number of candidate genes under the linkage peak, a family-based gene-wise study using famSKAT was performed (Wu et al., 2011), yielding four significant candidate genes, namely *DCAF7, POLG2*, *CEP95, and SMURF2* for 17q23.2, and five candidate genes, namely *RASGEF1A, HNRNPF, ANF487, CSTF2T, and PRKG1* for 10q11.21 (Figures 3A,B; Table 4). Two genes—*CEP95* (*p* = 0.000189) and *SMURF2* (*p* = 0.000271)—had the strongest support for gene-wise association.

**Table 4:** **Association of leukocyte telomere length and candidate genes under the significant linkage signals**.

**CHR**	**Gene**	**~cM**	Start (bp)^*^	**end (bp)**	# of SNPs^**^	**p_gene-wise_**
17	***17q23.2 (LOD = 2.52; HLOD = 4.77 ^&^)***					
	*DCAF7*	95.04–95.76	61,628,682	61,673,745	35	0.040309
	*POLG2*	96.11–96.76	62,466,058	62,493,829	12	0.031087
	*CEP95*	96.11–96.76	62,494,043	62,528,641	19	0.000189
	*SMURF2*	96.11–96.76	62,536,879	62,694,893	38	0.000271
10	***10q11.21 (LOD = 2.72; HLOD = 4.36 ^&^)***					
	*RASGEF1A*	67.54–68.01	43,682,162	43,859,398	108	0.011053
	*HNRNPF*	67.54–68.01	43,871,952	43,926,871	36	0.032890
	*ANF487*	67.54–68.01	43,904,919	44,050,715	63	0.010759
	*CSTF2T*	73.00–73.52	53,455,004	53,461,266	16	0.046672
	*PRKG1*	73.00–73.52	53,459,593	54,073,739	596	0.040813

* *HG19*.

** *Number of genotyped SNPs*.

& *HLOD based on all families*.

To identify variants that may contribute to variation in leukocyte telomere length in these genes, measured genotype analysis was performed for each SNP in the gene using SOLAR (Tables 5A, B). For 17q23.2, multiple SNPs in *CEP95* and *SMURF2* supported allelic association at *p* < E-5. We note that the results from the measured genotype analysis using SOLAR did not differ from those in the mixed linear model above. Because multiple contiguous SNP were associated with variation in leukocyte telomere length, haplotype analysis was performed. Table 5A shows that one contiguous haplotype G-T-T-T-G in *CEP95* and C-T-G-C-G-C-A-A-C-T in *SMURF2* was significantly associated with leukocyte telomere length (*p* < 0.0057 for transformed leukocyte telomere length). Due to low allele frequencies of risk variants, two haplotypes—the risk haplotype in black and the reference haplotype in white—were observed as shown in Table 5A. The mean leukocyte telomere length in haplotype carriers was shorter than that in non-carriers (5275.5 vs. 5329.4 base pairs, respectively). Similarly, for 10q11.21, a measured genotype analysis identified multiple variants in the *HNRNPF* gene (*p* < E-4) and the *PRKG1* gene (*p* < E-5).

**Table 5A:**
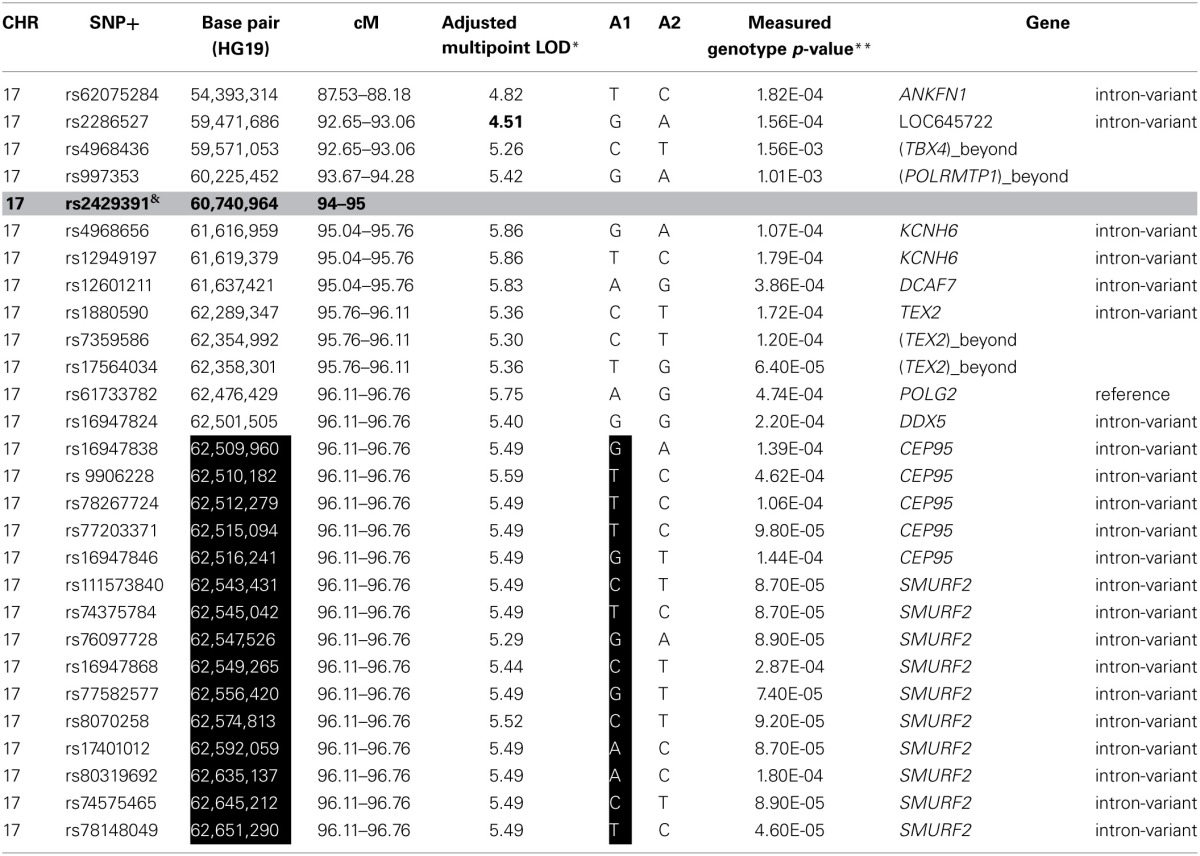
**SNP and haplotype association under the 17q23.2 linkage peak**.

^&^The linkage peak was located at rs2429391^*^−94 cM.

^*^Adjusted LOD at 94 cM in 6 linked families, adjusting for one SNP plus all previously mentioned covariates.

^**^Allelic association for each SNP using all subjects.

*Haplotype in CEP95 and SMURF2 are in black*.

*Rare haplotype frequency for G-T-T-T-G-C-T-G-C-G-C-A-A-C-T in CEP95 and SMURF2 is 0.0377*.

**Table 5B:** **SNP association under the 10q11.21 linkage peak**.

**CHR**	**SNP**	**Base pair (HG19)**	**cM**	Adjusted multipoint LOD^*^	**A1**	**A2**	Measured genotype *p*-value^**^	**Gene**	
10	rs117783414	29,005,814	56.01–56.52	9.15	T	C	4.17E-04	(*BAMBI*)_beyond	
**10**	**rs17500653^&^**	**35,535,597**	**63**	–				–	–
10	rs56080575	43,788,778	67.54–68.01	9.09	A	G	1.52E-04	(*RASGEF1A*)_beyond	–
10	rs2460535	43,821,380	67.54–68.01	**9.02**	G	A	4.33E-04	(*FXYD4*)_beyond	–
10	rs59383062	43,847,135	67.54–68.01	9.15	A	C	1.46E-04	(*FXYD4*)_beyond	–
10	rs10466239	43,849,827	67.54–68.01	9.47	T	C	5.62E-06	(*FXYD4*)_beyond	–
10	rs11814409	43,852,721	67.54–68.01	9.15	C	A	3.39E-04	(*FXYD4*)_beyond	–
10	rs80027918	43,881,183	67.54–68.01	9.69	A	G	1.91E-04	*HNRNPF*	reference
10	rs13376803	43,881,355	67.54–68.01	9.69	G	A	2.07E-04	*HNRNPF*	reference
10	rs10409	43,881,921	67.54–68.01	9.69	C	T	1.99E-04	*HNRNPF*	reference
10	rs 10899803	43,940,898	67.54–68.01	9.69		T	1.85E-04	*ZNF487P*	intron-variant
10	rs10899803	43,940,898	67.54–68.01	8.26	C	T	1.85E-04	*ZNF487P*	intron-variant
**10**	**rs2843562^&^**	**48,313,591**	**~72**						
10	rs1904017	53,853,542	73.00–73.52	8.45	G	A	3.39E-04	*PRKG1*	intron-variant
10	rs1904013	53,859,440	73.00–73.52	8.45	A	C	7.40E-05	*PRKG1*	intron-variant
10	rs60830257	53,867,534	73.00–73.52	8.45	A	G	6.90E-05	*PRKG1*	intron-variant
10	rs61448551	53,874,705	73.00–73.52	8.45	C	T	4.70E-05	*PRKG1*	intron-variant
10	rs16927026	53,887,029	73.00–73.52	8.45	G	A	6.50E-05	*PRKG1*	intron-variant
10	rs58118931	53,893,871	73.00–73.52	8.45	C	T	7.10E-05	*PRKG1*	intron-variant

& *The 2 linkage peaks (~63 and ~72 cM) were located near these SNPs*.

* *Adjusted mulitipoint LOD at 63 and 72 cM in 12 families, adjusting for one SNP plus all previoiusly adjusted coavariates*.

** *p-values for association for each SNP, adjusting for covariatesGene*.

### Confirmation of previous findings

Previously implicated genes (*TERC, ARPM1, MYNN, OBFC1,and ZNF729*) were examined to determine whether families ascertained based exceptional healthy aging also support allelic association for the same five reported SNPs in those genes. As shown in Table 6, 20 SNPs from the five genes were associated with leukocyte telomere length at *p*_nominal_ < 0.05. Of those, *TERC, MYNN*, and *OBFC1* remained significant at gene-wise *p* < 0.05 based on a permutation based test, and *MYNN* was significant at experiment- wise *p* < 0.05. However, with the exception of rs1317082 on *MYNN*, different variants were associated with leukocyte telomere length in the present study.

**Table 6:** **Association in previously reported genes from other studies**.

**Chr**	**Gene**	**SNP**	Position (bp)^*^	***N***	**Allele freq**	**Risk allele**	**Beta**	Nominal P^**^	**SNPwise-*P***	**Genewise-*p***	**Experiment-wise-*p***	**Role**
2	*(CXCR4)*	rs75157608	136,994,500	4276	0.0005	T	−0.96	0.04476	**0.025**	0.338	0.712	
**3**	***(TERC)***	**rs12638862**	**169,477,506**	**4278**	**0.2498**	**G**	**−0.0714**	**0.00676**	**<0.002**	**0.010**	0.374	
3	*(TERC)*	rs12630450	169,480,204	4274	0.2647	G	−0.0553	0.03325	**0.02**	0.060	0.864	
3	*ARPM1*	rs2068178	169,485,639	4278	0.0219	T	0.1843	0.02198	**0.038**	0.082	0.852	missense
3	*ARPM1*	rs9822885	169,486,144	4272	0.2637	G	−0.0565	0.03018	**0.02**	0.086	0.852	intron-variant
3	*ARPM1*	rs9860874	169,486,271	4277	0.2634	A	−0.0579	0.02613	**0.016**	0.066	0.810	intron-variant
3	*ARPM1*	rs9866776	169,487,651	4252	0.2619	A	−0.0571	0.0289	**0.016**	0.070	0.826	reference
3	*(MYNN)*	rs3821383	169,489,946	4263	0.2635	C	−0.0594	0.0228	**0.013**	0.089	0.768	
**3**	***MYNN***	**rs10936599**	**169,492,101**	**4276**	**0.2289**	**T**	**s−0.0944**	**0.000483**	**<0.002**	**0.002**	**0.044**	**nc-transcript-variant**
**3**	***MYNN***	**rs1317082^&^**	**169,497,585**	**4270**	**0.2292**	**G**	**−0.0964**	**0.000372**	**<0.002**	**0.002**	**0.037**	**intron-variant**
10	*(OBFC1)*	rs2902638	105,636,989	4276	0.2572	C	−0.0604	0.0198	**0.017**	0.190	0.534	
10	*OBFC1*	rs10748858	105,639,514	4277	0.4322	G	0.0518	0.02305	**0.012**	0.128	0.390	reference
10	*OBFC1*	rs11191841	105,639,611	4276	0.4828	C	−0.0575	0.01105	**<0.002**	0.059	0.204	reference
**10**	***OBFC1***	**rs7100920**	**105,640,978**	**4277**	**0.4755**	**T**	**−0.0598**	**0.00806**	**<0.002**	0.051	0.178	**reference**
**10**	***OBFC1***	**rs2067832**	**105,643,134**	**4268**	**0.4752**	**T**	**−0.0627**	**0.005524**	**<0.002**	**0.037**	0.130	**intron-variant**
10	*OBFC1*	rs11598840	105,645,181	4270	0.1006	A	0.0767	0.04448	0.064	0.522	0.918	intron-variant
10	*OBFC1*	rs4918069	105,654,391	4274	0.2763	G	−0.0553	0.02839	**0.018**	0.256	0.638	intron-variant
10	*OBFC1*	rs77987791	105,670,702	4278	0.0007	T	1.111	0.0375	**0.0472**	0.395	0.832	intron-variant
19	*ZNF729*	rs115647405	22,483,912	4278	0.0003	T	2.339	0.01871	**0.01**	0.224	0.532	intron-variant
19	*ZNF729*	rs76712090	22,486,056	4267	0.0127	T	−0.2122	0.03684	**0.0312**	0.405	0.823	intron-variant

* *HG19*.

** *Nominal p-values are presented for each SNP; however, SNPs with empirical p-value <0.05 are shown in bold*.

*Only the genotyped SNPs are presented*.

& *This SNP was previously reported*.

## Discussion

We identified genetic factors that contribute to variation in leukocyte telomere length in families that were selected for exceptional longevity who also experienced healthy aging. The genome wide association analysis revealed that *DKK2* and *PAPSS1* on 4q25 are strong candidate genes (p4.7 < E-8) that may contribute to variation in leukocyte telomere length, and that several other genes may also be involved. In addition, two novel loci—17q23.2 (HLOD = 4.77) and 10q11.21 (HLOD = 4.36)—had HLOD scores exceeding 3. From the multiple candidate genes present under the linkage peaks, we identified nine genes that were significantly associated with leukocyte telomere length at gene-wise level, which include four genes in 17q23.2 (specifically *DCAF7, POLG2*, *CEP95, and SMURF2*) and five genes in 10q11.21 (specifically, *RASGEF1A, HNRNPF, ANF487, CSTF2T, and PRKG1*). Among these genes, *CEP95* and *SMURF2* in 17q23.2 had one contiguous novel haplotype that was significantly associated with leukocyte telomere length. In 10q11.21, SNPs in *HNRNPF* and *PRKG1* were also associated. Further, we confirmed association between leukocyte telomere length and *TERC, MYNN, and OBFC1*. Our approach of combining association and linkage analyses has identified a set of novel genes that contribute to variation in leukocyte telomere length in families with exceptional longevity characterized by healthy aging. However, it is difficult to decipher the role of these candidate genes in cellular aging since differential cellular aging can arise from the fundamental processes such as cell death, or by influencing the disease processes in age related diseases. Here we discuss several genes that may contribute to biological cellular aging as measured by leukocyte telomere length.

The strongest GWAS signal was observed in a set of five SNPs located in 4q25, and this finding refines and extends the earlier report of linkage peak at D4S1564 (MLS = 3.65, *p* = 0.044; 108,376,510-108,376,856 bp) based on 137 sibships selected for longevity (Puca et al., 2001). These SNPs are located between the *DKK2 and PAPSS1* genes, but one of the SNP localizes to *DKK2*. Because of limited work done on these genes, their role in cellular aging is unclear. The *DKK2* gene is believed to be involved in embryonic development, and interacts with LDL-receptor related protein 6 (LDL6). Based on biological similarity, it may be involved in bone diseases, cancer and Alzheimer disease in adults (Magrane and Consortium, 2011). The *PAPSS1* gene encodes a trypsinogen that is a member of the trypsin family of serine proteases. Mutations in this gene are associated with hereditary pancreatitis. To date, however, both genes have not been implicated in common diseases in humans.

*TRDMT1*, Homo sapiens tRNA aspartic acid methyltransferase 1, on 10p13, is involved in DNA methylation. Because of its role in methylation, Halaschek-Wiener et al. (2009) considered it to be a candidate gene for healthy aging and sequenced 47 individuals who survive to age 85 or older without any major age-related diseases. This exploratory study observed that *TRDMT1* and *SIRT3* had the highest frequency of variants; however, the role of *TRDMT1* in aging is unclear because allele frequencies for SNPs in this gene were not compared to those in controls. Another candidate gene, *SYT16* on 14q23.2 from the GWAS, was reported to be involved in *trafficking* and exocytosis of secretory vesicles in non-neuronal tissues. Mosing et al. (2010) reported that one of the SNPs in the gene was associated with self-rated health.

Our genome wide linkage analysis revealed several genes that would have been missed by the GWAS, had we restricted our analysis to genes that meet the strict genome wide significance threshold. Genome wide linkage analysis followed by gene-wise association analysis identified *CEP95* (Centrosomal protein 95 kDa) and *SMURF2* (SMAD specific E3 ubiquitin protein ligase 2) as promising candidate genes. *SMURF2* is shown to be involved in regulation of neuronal and cell polarity, induction of cellular senescence, and tumor suppression (Blank et al., 2012), suggesting its potential role in cellular aging. On the other hand, very little is known about *CEP95*. However the shared haplotype encompassing these two genes is a strong candidate region for sequencing. In addition, *KCNH6* (potassium voltage-gated channel, subfamily H (Ether-A-Go-Go-Related), member 6) belongs to a class of voltage-gated ion channels and is believed to be involved in regulating release of neurotransmitters, controlling heart rate, secretion of insulin, neuronal excitability, etc. (http://genecards.org).

Among several genes on 10q11.21 that showed significant gene-wise association, including *RASGEF1A*, *HNRNPF, ANF487, CSTF2T*, and *PRKG1*, *HNRNPF* and *PRKG1* are candidate genes of interest. *HNRNPF* is involved in multiple regulatory pathways, and it has been associated with late onset Alzheimer disease, (Grupe et al., 2006) modulate neuronal viability (Boucher et al., 2002), and is also reported to be associated with cancers. *PRKG1* regulates cellular platelet activation and adhesion, contraction of smooth muscles, cardiac function, and other processes involved in several functions association with central nervous system function, such as axon guidance, hippocampal and cerebellar learning, etc. These genes may be biologically relevant to healthy aging, requiring further examination.

The present study confirmed the genes that were previously reported to be associated with leukocyte telomere length (Vasa-Nicotera et al., 2005; Andrew et al., 2006; Mangino et al., 2009; Codd et al., 2010; Levy et al., 2010; Mangino et al., 2012). Specifically, we found at least one SNP in *TERC, ARPM1, MYNN, OBFC1*, and *ZNF729* to be *nominally* associated with variation in leukocyte telomere length; however, *TERC, MYNN*, and *OBFC1* were significantly associated with leukocyte telomere length based on a permutation test at the gene-wise level. Moreover, *MYNN* was significant at experiment-wise level. For these genes, different variants were significantly associated with leukocyte telomere length, except for *MYNN*, where rs1317082 (*p* = 0.000372) (Mangino et al., 2012) was previously reported.

Several possibilities may explain the differences in findings across studies, including selection, leukocyte telomere length measurements, differences in the distribution of risk factors, etc (Aviv et al., 2006; Christensen et al., 2006; Armanios and Blackburn, 2012; Sanders et al., 2012). First, cellular aging as measured by leukocyte telomere length is likely to be a complex trait. As such, a different set of genetic and environmental risk factors can influence variation in leukocyte telomere length in different cohorts. The LLFS cohort was sampled to recruit families with strong evidence for familial longevity, and this cohort appears to be relatively healthier than other elderly cohorts (Newman et al., 2011). Previously, Newman et al. (2011) showed that compared with other large epidemiologic cohorts (including Cardiovascular Health Study, the Framingham Heart Study, and the New England Centenarian Study), the prevalence of diabetes, chronic pulmonary disease and peripheral artery disease was lower in LLFS family members and the levels of biomarkers (e.g., pulse pressure, triglycerides, HDL, and gate speed) were more favorable. In addition, the heritability estimates for leukocyte telomere length in this cohort ranged from 0.47 for the older proband generation to 0.85 for the relatively younger offspring generation with an overall estimate of 0.54. These estimates are comparable to those observed in the meta-analysis based on 19,713 subjects by Broer et al. (2013) which observed an overall heritability of 0.70, with 0.62 for the Netherlands Twin Registry and Queensland Institute Medical Research Twin study and 0.86 for the Leiden Longevity Study. However, given the difference in recruitment for LLFS compared with other studies, it is likely to yield a different set of candidate genes than in other datasets that have been ascertained for familial aggregation of heart disease or a random set of twins, for example. Moreover, there likely to exist substantial differences in life style or the distribution of environmental risk factors in these families selected for familial longevity compared with cohorts recruited based on diseases of interest. With the exception of age, sex, Caucasian ancestry, and atherosclerosis (Sanders and Newman, 2013), the strength and direction of association between leukocyte telomere length and risk factors are equivocal. Thus, to minimize the influence of these risk factors on allelic association, the present study adjusted for potential confounders, including age, sex, education, site, smoking, alcohol consumption, marital status, a history of heart disease, and one principle component because they were significantly associated with leukocyte telomere length in the LLFS dataset. Second, the telomere assays used across studies vary (Sanders and Newman, 2013). This study measured leukocyte telomere length using the quantitative PCR method (T/S ratio) (Cawthon, 2002; Cawthon et al., 2003; Honig et al., 2012), rather than the terminal restriction fragment method (TRF) (Aviv et al., 2011). The impact of methodological difference on the genetic findings is likely to be minimal. Studies that compared these two methods showed that the T/S ratio method measures only “canonical” TTAGGG telomere repeats, while the TRF method derives a “telomere” measurement including telomere-adjacent non-canonical sequences. However, over a wide-range, T/S measurements linearly relate to TRF measurements, as shown by many investigators, including Cawthon et al. (2003), Honig et al. (2012), and Aviv et al. (2011). This study supports comparability of the two methods by confirming the previously reported genes. Lastly, because the present study ascertained healthy families selected for familial longevity, there exist very few studies with such extreme sampling exist. Therefore, the present study lacks extensive replication since reported associations vary widely depending on study design (e.g., case-control vs. family based or randomly selected samples vs. extreme samples, etc.).

In sum, the present study identified novel variants in several genes in three loci—4q25, 17q23.2, and 10q11.21—that may contribute to variation in leukocyte telomere length in families with exceptional longevity. The findings from this study may facilitate identification of genes that may better explain how cells age, thereby enhancing our understanding of aging mechanisms.

### Conflict of interest statement

The authors declare that the research was conducted in the absence of any commercial or financial relationships that could be construed as a potential conflict of interest.
